# Effect sizes of aerobic exercise and cognitive training interventions on blood biomarkers over 6 months in the ACT Trial

**DOI:** 10.1002/alz70856_102229

**Published:** 2025-12-25

**Authors:** Danni Li, Binchong An, Dereck L. Salisbury, Feng Vankee Lin, Fang Yu

**Affiliations:** ^1^ University of Minnesota, Minneapolis, MN, USA; ^2^ University of Minnesota, Twin Cities, Minneapolis, MN, USA; ^3^ Stanford University, Stanford, CA, USA; ^4^ Arizona State University, Phoenix, AZ, USA

## Abstract

**Background:**

It remains unclear whether blood biomarkers of Alzheimer's disease are surrogate endpoints for non‐pharmacological interventions. The ACT Trial blood biomarker study aimed to evaluate neuropathological, neurotrophic, and neuroinflammation biomarkers as surrogate outcomes in older adults with mild cognitive impairment (MCI). This presentation assessed whether any ACT intervention group (ACT, cycling only, SOP only) had significant changes in biomarkers compared to the attention control group over 6 months. Also, we evaluated the effect sizes of interventions on blood biomarkers over 6 months.

**Method:**

We collected blood samples from 95, 81, 76, 53, and 39 ACT Trial participants (*n* = 344) over five time points (baseline, 3, 6, 12, and 18 months). We biochemically analyzed neuropathological biomarkers (plasma Ab_42_, Ab_40_, NfL, *p*‐Tau 217), neurotrophic biomarkers (plasma IGF‐1, IGFBP‐3, and serum free‐BDNF), and neuroinflammation biomarkers (plasma GFAP and clusterin). We used mixed effects models to examine whether any intervention group was associated with a significant difference in biomarkers (3 or 6 months compared to baseline [i.e., 0‐3 m or 0‐6m]) compared to the control group. We also calculated effect size estimates (Cohen's d) for within‐group differences in biomarkers.

**Result:**

The mean (SD) age of the 102 participants with MCI was 73.83 (6.07) years old, 45.10% (46/102) female, and 34.31% (35/101, 1 missing) APOE4 carriers. There were no significant differences of 0‐3 m or 0‐6 m in biomarkers of any intervention group compared to that of the control. Most biomarkers had small within‐group Cohen's d (less than 0.2) except 0‐6 months plasma *p*‐Tau 217 (d=0.27) and 0‐6 m serum free‐BDNF (d=0.71) in the control group.

**Conclusion:**

We did not find a significant effect of interventions on blood biomarkers. Plasma *p*‐Tau 217 and serum free‐BDNF had small and moderate effect sizes to detect changes in the control group of older adults with MCI over 6 months, respectively.

